# Apoptosis of Platelets Inhibited By Herba Sarcandrae Extract through the Mitochondria Pathway

**DOI:** 10.1155/2018/1956902

**Published:** 2018-11-18

**Authors:** Xiaoqin Zhu, Yiling Jiang, Qin Zheng, Aiping Zhang, Ling Shi, Lemin Xia, Meihong Luo

**Affiliations:** ^1^Department of Hematology, Shanghai Baoshan Hospital of Integrated Traditional Chinese and Western Medicine (Baoshan Branch of Shuguang Hospital Affiliated to Shanghai University of Traditional Chinese Medicine), Shanghai 201999, China; ^2^Department of Hematology, Shuguang Hospital Affiliated to Shanghai University of Traditional Chinese Medicine, Shanghai 200021, China

## Abstract

The purpose of the present study is to decode the underlying mechanism of Herba Sarcandrae that indicated antipurpuric effect and to unveil one of its primary components, flavonoids, which play an important role. An immune mediated bone marrow failure (BMF) model in mouse was established by infusion thymus suspension cells after radiation* in vivo. *Platelets isolated* in vitro *were prepared from normal mice and BMF mice, respectively. The expressions of PS, P-selectin, PAC-1, Bax, Bad, Bid, and caspase-9 were examined by flow cytometry, and alteration of morphology of platelets under different conditions was observed. Our results indicated that the number of platelets was increased by addition of total flavonoids, and some of apoptotic markers such as Bax, Bad, Bid, and Caspase-9 were downregulated. In addition, the phosphatidylserine (PS) exposure on platelets was inhibited by total flavonoids, and the expressions of PAC-1 and P-selectin were decreased. In conclusion, it is suggested that the total flavonoids of Herba Sarcandrae may inhibit the excessive platelet apoptosis through mitochondrial pathway. In addition, activation of platelets may be also involved in mediating apoptosis of platelets.

## 1. Introduction

Herba Sarcandrae (HS), one of the traditional Chinese herbal medicines, has been widely employed to treat rheumatoid arthritis, bruise, bone fractures, purpura, and malignant solid tumors, relying on its main components such as rocaglamides, aglain, bisamides, flavonoids, tetracyclic triterpenoids, coumarin, and volatile oil. Herba Sarcandrae is the ground part or the dry whole grass of the Sarcandra glabra, a perennial herb of Chloranthaceae [1]. It is a registered medicinal herb recorded in the 2015 version of “the People's Republic of China Pharmacopoeia”. The pharmacological effects have been reported in treating idiopathic thrombocytopenic purpura (ITP) and secondary thrombocytopenic purpura (STP). The long-term therapeutic efficacy of TF on ITP is better than that of glucocorticoids, because fewer adverse side effects are observed for TF than those for glucocorticoids; e.g., neutrophils are decreased by glucocorticoids but not by TF, and immunity inhibition is induced by glucocorticoids but not by TF. HS has been employed as alternative therapy to help recovery of STP induced by chemotherapy on tumors or immunity inhibition from allograft organ transplantation. By the virtue of Chinese Traditional Medical Theory, HS is helpful to facilitate circulation to remove blood stasis, diminish swelling and lump, and cool blood for hemostasis that is intended to treat blood-heated bleeding and various thrombocytopenia [2]. Total flavonoid (TF) is the major effective ingredient from Herba Sarcandrae (HS) against purpura.

Total flavonoids are primarily components (9 elements) from HS ethanol-extraction (HS represents HS extraction in the whole text). However, most of the efficacy of HS is heavily dependent on its mixtures of hydrophobic extraction instead of any single elements. In the previous study, it was observed that the proliferation of bone marrow megakaryocytes is facilitated by HS in mice and the number of platelets is increased [3]. Previously, a thrombocytopenia model is established in mice via intraperitoneal injection (i.p.) with cytarabine 100 mg/kg for successive 7 days, and the number of leukocytes and platelets is significantly increased by the total flavonoids of Herba Sarcandrae [4]. It was observed that the total flavonoids had little effect on the myelosuppression induced by chemotherapeutic drugs, but the total flavonoids at 200 mg/kg significantly protected platelet count from cyclophosphamide [5]. Although the total flavonoid is one of the effective constituents from HS in treating bleeding, the underlying mechanism remains unclear.

The platelet plays an important role in maintaining hemostasis. Either the quantity or the quality of platelets is changed, which leads to the disorder of hemostasis. Even without nuclei, apoptosis of platelets was also observed via intrinsic and extrinsic apoptotic pathways in recent years [6]. Among two apoptotic pathways, the intrinsic pathway (i.e., mitochondria pathway) is thought to be an important way to mediate the platelets' apoptosis [7]. The mechanism of the mitochondrial pathway includes depolarization of mitochondrial transmembrane potential (ΔΨm), which appears as the early manifestation and characteristic change of apoptosis. A variety of mitochondrial proteins transfer to cytoplasm, and those proteins play roles in regulating the platelets' apoptosis. The apoptotic precursor proteins including Bax, Bak, and Bid are expressed, activated, and translocated into mitochondria; the further release of cytochrome c is induced by the opening of mitochondrial permeability transition pore (MPTP); the mitochondrial external downstream changes, for example, caspase-9 activation and phosphatidylserine (PS) exposure [8] caused by some chemical stimulations [9, 10]. It has been shown that the immune-induced bone marrow failure mouse model has thrombocytopenia, decrease of cysteine-containing aspartate-specific proteases (caspase)-8 and caspase-3 and change of other apoptotic markers. It is indicated that the pathogenesis of thrombocytopenia is closely associated with platelet apoptosis [11]. One of the mechanisms of cyclosporine A (CSA) in the treatment of thrombocytopenia is to inhibit the mitochondrial pathway-mediated platelets apoptosis, thereby increasing platelet survival time [12]. However CSA is difficult to use for acute bleeding treatment because of its long onset time.

Our previous study has found that the platelet apoptosis mediated by mitochondrial pathway in immune-induced bone marrow failure resulted in abnormal coagulation and bleeding [13, 14]. In this study,* in vivo* and* in vitro *experiments were conducted to investigate whether the total flavonoids of Herba Sarcandrae could inhibit the platelets apoptosis by regulating the mitochondrial pathway.

## 2. Materials and Methods

### 2.1. Antibodies and Reagents

The monoclonal antibodies against Bax, Bad, Bid, and cysteine caspase-9 were obtained from Santa Cruz Biotechnology (Santa Cruz, CA, USA). The monoclonal antibodies against P-selectin, phosphatidylserine (PS), fluorescein isothiocyanate-labeled platelets activated complex-1 (FITC-PAC-1), and hydroxy cyanide chlorophenylhydrazone (CCCP) were purchased from Sigma (Saint Louis, Missouri, USA). The cyclosporine (CSA) capsule was purchased from Novartis German Pharmaceutical Co., Ltd., Batch number: S0408, 25 mg x 50 granule/box. The 4 mg/ml CSA solution is configured by sterile saline.

### 2.2. Preparation of Total Flavonoids of Herba Sarcandrae and Cyclosporine

**Figure 1:**
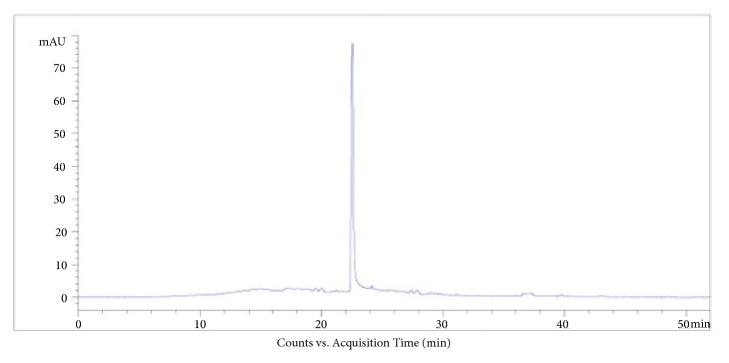
**The chromatographic fingerprint of the total flavonoids of Herba Sarcandrae by high performance liquid chromatography.** The retention time of hydrolysate is 21 minutes.

Herba Sarcandrae was provided by our hospital pharmacy. The total flavonoids of Herba Sarcandrae were prepared by macroporous resin HPD400. The process conditions for total flavonoids separation were in accordance with methods reported by Xu et al.[15]. The chromatographic fingerprint was established by high performance liquid chromatography and listed as Figure 1. Cyclosporine (S0408, 25 mg × 50 tablets, Novartis Pharma, Freiburg Area, Germany) was added to 4 mg/mL solution with sterile saline. Lavage solution is then diluted to 0.1 mL per 10 grams of mouse weight.

### 2.3. Animals and Treatments

The study was approved by the Ethics Committee of Laboratory Animals of Integrated Chinese and Western Medicine Hospital of Baoshan District, Shanghai, China. Clean C57BL/6 mice (half male and female, 8-12 weeks, 20 ± 2 g body weight) were provided by Shanghai SLAC Laboratory Animal Co., Ltd., and raised in the environment of room temperature (22 ± 2)°C, humidity 55%, 12 h light and dark reared, and free food and water. The animal license number is SCXK (Shanghai) 2012-0002. The proper housing, feeding, and care and all interventions related to the animal welfare were carried out in compliance with the stipulations of Regulations for the Administration of Affairs Concerning Experimental Animals (China).

The bone marrow failure mouse model was established according to the methods described by Zhao et al. [16]. Briefly, DBA/2 mice were sacrificed and soaked in 75% ethanol for 5min. The thymus was removed after routine disinfection and a small amount of saline was used to wash away the blood. The thymus was gently ground and filtered with nylon filter. Single cell suspension was made through 4 gauge needles. Cell viability was tested by trypan blue staining (up to 95% viable cells). The single cell suspension was adjusted to the desired concentration after cell counting. A total of 1 × 10^6^ cells were then administered through the tail vein of a C57BL/6 mouse for 4 h after exposure to ^60^Co-*γ* radiation [5.5Gy (1.1 Gy/min × 5 min)]. After 3 days, the mice tail vein blood was taken and the blood routine test was performed by the automatic blood cell analyzer. The complete decrease of the blood cells suggests the success of the bone marrow failure mouse model.

A total of 40 mice were randomly divided into four groups with 10 mice in each group: normal control group, model group, CSA treatment group, and flavone treatment group. The mice in model group, CSA treatment group, and flavone treatment group received bone marrow failure modeling. Besides, mice in CSA treatment group were orally gavaged with 0.027 g/kg cyclosporine daily, while mice in flavone treatment group were orally gavaged with 0.2 g/kg total flavonoids daily. Same amount of saline was given to the mice in normal control group and model group. All mice were orally gavaged once a day for 3 consecutive days. On the third day of the experiment, the mice were injected with 10 % hydrochloride 0.005 ml/g for anesthesia. Blood samples were collected from the caudal venous plexus for the blood routine test and the preparation of the washed platelets. The platelet count was determined by automatic blood cell analyzer.

### 2.4. Preparation of Washed Platelets

Briefly, 0.7 ml venous blood was anticoagulated with ACD (2.5 % sodium citrate, 2 % glucose, 1.5 % citric acid) by 1:7 and centrifuged at 1300 RPM for 20 min to get the platelet rich plasma (PRP). The PRP was further centrifuged at 2500 RPM for 20 min and the supernatant was discarded. The platelets were washed with CGS buffer (0.123 M sodium chloride, 0.033 M glucose, 0.013 M sodium citrate, pH 6.5) and resuspended with Tyrode's buffer (2.5 mMHepes, 150 mM sodium chloride, 2.5 mM potassium chloride, 1 mM calcium chloride, 1 mM magnesium chloride, 12 mM sodium bicarbonate, 5.5 mM glucose, pH 7.4). The platelet number was counted by cell-count boards. The number of platelets was adjusted to 3×10^8^/ml in the suspension and the platelet suspension was allowed to stand at room temperature for 60 min.

### 2.5. Induction of the Washed Platelets In Vitro

The washed platelets prepared from the mice in normal control group and model group were used for the* in vitro *study. There were 7 different groups, namely, model control group (BMF group), normal control group, flavone group (washed platelets were incubated with 250 *µ*g/ml total flavonoids), apoptosis inducer group (Carbonyl cyanide chloride group or CCCP group; 100 *µ*g/ml CCCP), apoptosis inhibitor group (CSA group; 10 *µ*g/ml CSA ), CCCP + flavone group (100 *µ*g/ml CCCP and 250*µ*g/ml total flavonoids), and CSA + flavone group (10 *µ*g/ml CSA and 250*µ*g/ml total flavonoids). The platelets prepared from normal mice were used for the normal control group, CCCP group, and CCCP + flavone group. The platelets prepared from BMF model mice were used for the BMF control group, flavone group, CSA group, and CSA + flavone group.

### 2.6. Flow Cytometry

The procedure of flow cytometry was employed by the previous study [17]. Briefly, the expressions of PS, P-selectin, PAC-1, Bax, Bad, Bid, and caspase-9 were detected by flow cytometry. Washed platelets (3×10^8^ /ml) from different groups were incubated at 37°C for 5 h. Annexin V binding buffer was then mixed with pretreated platelets and FITC-annexin V at a ratio of 50: 10: 1. Samples were mixed gently, incubated at room temperature for 15 min in the dark, and then subjected to flow cytometry to detect the PS externalization. For P-selectin surface expression detection, the platelets were incubated with P-selectin specific antibody SZ51 at room temperature for 30 min, then incubated with FITC-GAM in the dark at room temperature for 30 min, and subjected to flow cytometry analysis. In PAC-1 binding assay, the platelets were incubated with FITC-labeled soluble PAC-1 and incubated at room temperature for 20 min in the dark. The treated platelets were fixed with 1% paraformaldehyde, further incubated at 4°C in the dark for 30 min. Then the treated samples were subjected to flow cytometry to detect the PAC-1 binding ability. To detect the activation of Bax, Bad, Bid, and caspase-9, 50 *µ*l antibodies and 50 *µ*l washed platelets were incubated at 37°C in the dark for 20 min and then subjected to flow cytometry.

### 2.7. Platelet Ultrastructure

The protocol was adopted from the previous study reported by Ding et al. [18]. Changes in the morphology and number of platelet apoptosis from various* in vitro *study groups were recorded by inverted phase contrast microscope (Leica Instruments, Wetzlar, Germany).

### 2.8. Statistical Analysis

Data are shown as means ± standard deviation (SD). The statistical difference among different groups was further determined by ANOVA. A* p*-value less than 0.05 was considered significant. All the data were analyzed by SPSS19.0 (SPSS, Chicago, Illinois, USA).

## 3. Results

### 3.1. The Number of Platelets Count in Circulation Was Increased by the Total Flavonoids In Vivo

**Figure 2:**
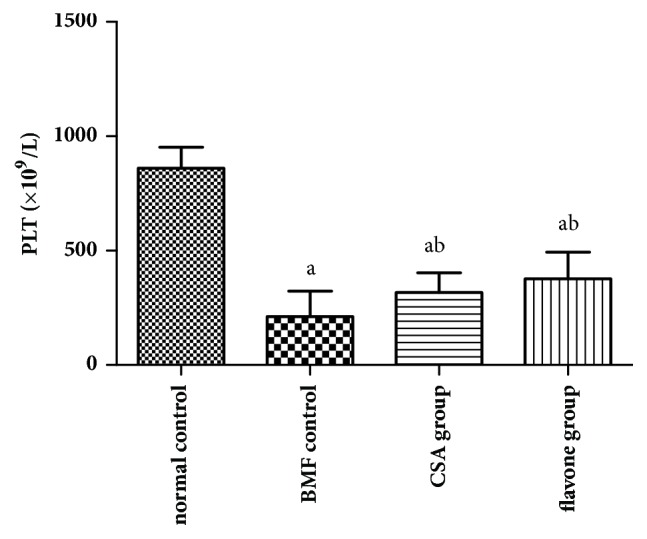
**Effect of total flavonoids on the platelets count* in vivo*. **Total flavonoids (0.2 g/kg) were given to the mice in flavone group. Mice in CSA group were given 0.027 g/kg cyclosporine daily. All mice were intragastrically administered once a day for 3 consecutive days. The platelet count was determined by automatic blood cell analyzer. ^a^*p*< 0.05 compared with the normal control group; ^b^*p*< 0.05 compared with the BMF group.

Compared with the normal control group, the platelets number of the model control group was decreased significantly. Compared with the model control group, the platelets number of the CSA group and the flavone group was increased significantly. Therefore, both the CSA and the total flavonoids can increase the platelets number in bone marrow failure mice (Figure 2).

### 3.2. The PS Exposure Was Inhibited by the Total Flavonoids In Vivo

**Figure 3:**
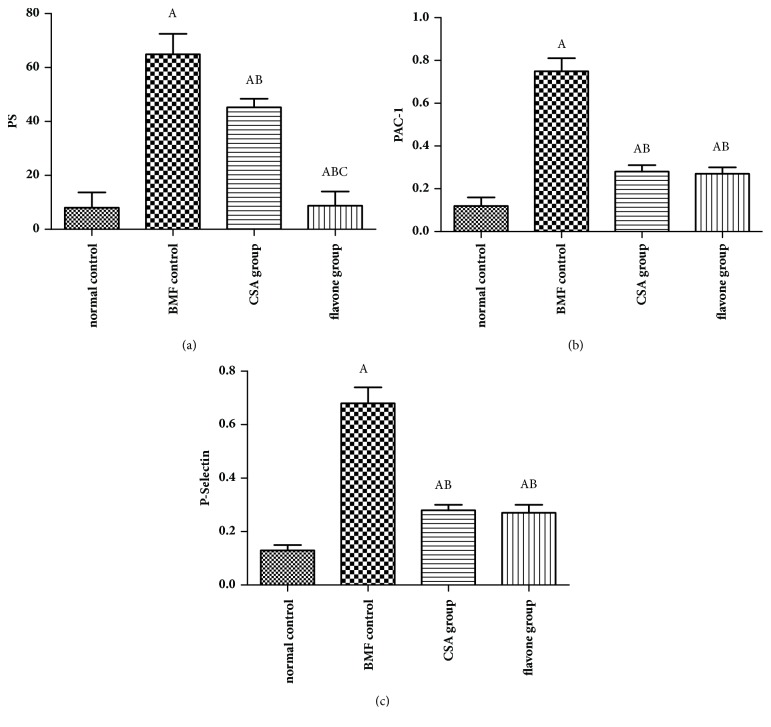
**The expressions of PS, PAC-1, and P-selectin in platelets. **Total flavonoids (0.2 g/kg) were given to the mice in flavone group. Mice in CSA group were given 0.027 g/kg cyclosporine daily. All mice were intragastrically administered once a day for 3 consecutive days. The expression levels of PS (a), PAC-1 (b), and P-selectin (c) were determined by flow cytometry. ^A^*p*< 0.05 compared with the normal control group; ^B^*p*< 0.05 compared with the BMF group; ^C^*p*< 0.05 compared with the CSA group.

Compared with the normal control group, the PS exposure of the model control group was increased significantly. Compared with the model control group, the PS exposure of CSA group and the flavone group was decreased significantly. Compared with the CSA group, the PS exposure of the flavone group was decreased significantly. Therefore, both the CSA and the total flavonoids could inhibit the PS exposure and the excessive platelets apoptosis effectively in the bone marrow failure mice model. The total flavonoids worked even better than CSA (Figure 3(a)).

### 3.3. The PAC-1 Binding of the Circulating Platelets Was Inhibited by the Total Flavonoids In Vivo

Compared with the normal control group, the PAC-1 binding of the model control group was increased significantly. Compared with the model control group, the PAC-1 binding of the CSA group and the flavone group was decreased significantly. No significant difference of the PAC-1 binding was found between the CSA group and the flavone group. Therefore, both the CSA and the total flavonoids could inhibit the PAC-1 binding in the bone marrow failure mice model (Figure 3(b)).

### 3.4. The P-Selectin Expression of the Circulating Platelets Was Inhibited by the Total Flavonoids In Vivo

Compared with the normal control group, the P-selectin expression of the model control group was increased significantly. However, compared with the model control group, the P-selectin expression in the CSA group and the flavone group was decreased significantly. No significant difference of the P-selectin expression was found between the CSA group and the flavone group. Therefore, both the CSA and the total flavonoids could inhibit the P-selectin expression in the bone marrow failure mice model (Figure 3(c)).

### 3.5. The Expressions of Bax, Bad, Bid, and Caspase-9 Were Induced by the Total Flavonoids In Vivo

**Figure 4:**
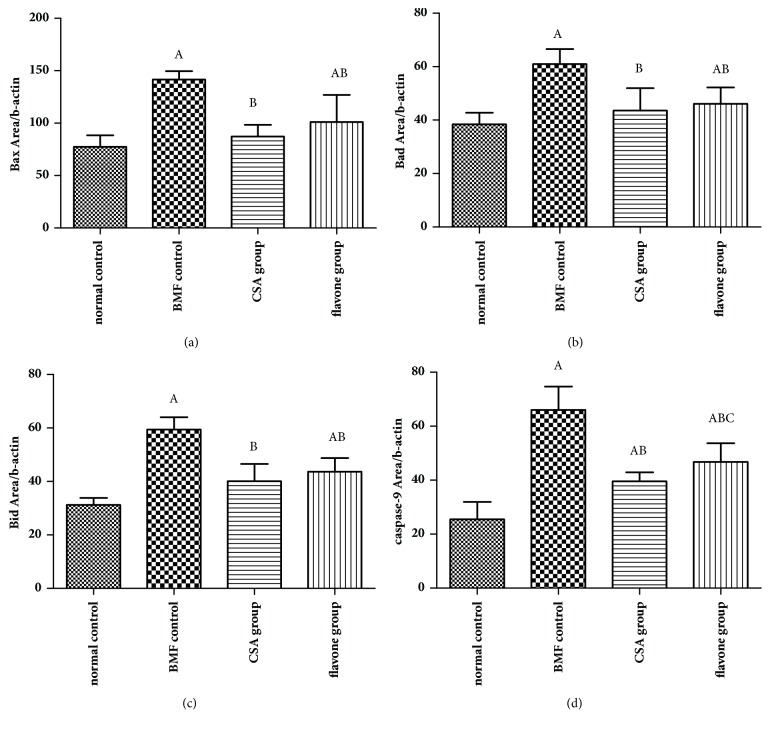
**The expressions of Bax, Bad, Bid, and Caspase-9 in platelets. **Total flavonoids (0.2 g/kg) were given to the mice in flavone group. Mice in CSA group were given 0.027 g/kg cyclosporine daily. All mice were intragastrically administered once a day for 3 consecutive days. The expression levels of Bax (a), Bad (b), Bid (c), and Caspase-9 (d) in platelets were detected by flow cytometry. ^A^*p*< 0.05 compared with the normal control group; ^B^*p*< 0.05 compared with the BMF group; ^C^*p*< 0.05 compared with the CSA group.

As shown in Figure 4, compared with the normal control group, the expressions of Bax, Bad, Bid, and caspase-9 of the model control group were increased significantly. Compared with the model control group, the expressions of Bax, Bad, Bid, and caspase-9 of the CSA group and the flavone group decreased significantly. What is more important, the caspase-9 expression level of the flavone group was much higher than that of the CSA group. It was indicated that the total flavonoids inhibited the excessive platelets apoptosis through the mitochondria pathway.

### 3.6. Effect of Total Flavonoids on Platelet Count In Vitro

**Figure 5:**
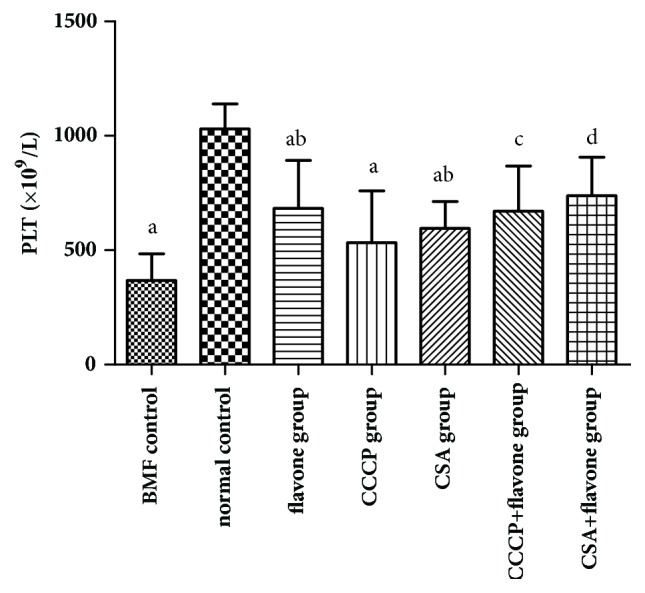
**The platelet count of mice in different* in vitro* study groups. **The washed platelets were prepared and treated with different drug interventions. The platelets prepared from normal mice were used for the normal control group, CCCP group, and CCCP + flavone group. The platelets prepared from BMF model mice were used for the BMF control group, flavone group, CSA group, and CSA + flavone group. The changes of platelet count were measured by flow cytometry. ^a^*p*< 0.05 compared with the normal control group; ^b^*p*< 0.05 compared with the BMF group; ^c^*p*< 0.05 compared with the CCCP group; ^d^*p*< 0.05 compared with the flavone group.

Compared with normal control group, the platelet number of mice in model control group and CCCP group was significantly reduced. The platelet count of mice in flavone group and CSA group was significantly higher than that in the model control group. Compared with the CCCP group, the platelet number of mice in CCCP + flavone group was increased. Compared with the flavone group, the platelet number of mice in CSA + flavone group was slightly increased. Therefore, the treatment of apoptosis inhibitor CSA and the total flavonoids could effectively improve the platelet count (Figure 5).

### 3.7. Effect of Total Flavonoids on PS Exposure In Vitro

**Figure 6:**
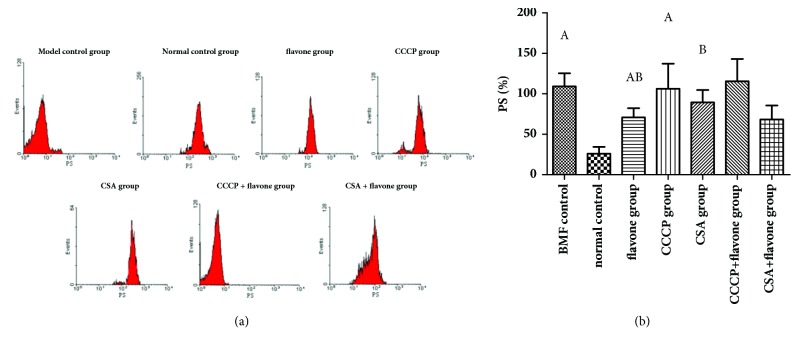
**The expressions of PS in different* in vitro* study groups. **The washed platelets were prepared and treated with different drug interventions. (a) Representative result of flow cytometry; (b) quantitation of the expression of PS of mice in different groups. ^*A*^*p*< 0.05 compared with the normal control group; ^B^*p*< 0.05 compared with the BMF group; ^C^*p*< 0.05 compared with the CCCP group; ^D^*p*< 0.05 compared with the flavone group.

Compared with the normal control group, the PS exposure of the model control group and CCCP group was significantly increased. Compared with the model control group, the PS exposure of the flavone group and CSA group was decreased significantly, but was still higher than that of the normal control group. Compared with the CCCP group, the PS exposure of the CCCP + flavone group was not changed significantly. It is indicated that the total flavonoids and the CSA inhibited PS exposure (Figure 6).

### 3.8. Effect of Total Flavonoids on the Expressions of PAC-1 and P-Selectin In Vitro

**Figure 7:**
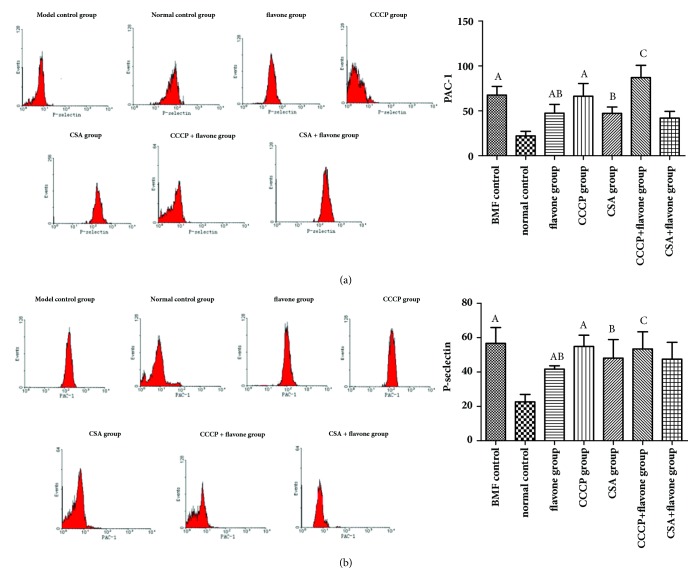
**The expressions of PAC-1 and P-selectin in different* in vitro* study groups. **The washed platelets were prepared and treated with different drug interventions. The expressions of PAC-1 (a) and P-selectin (b) were detected by flow cytometry. ^A^*p*< 0.05 compared with the normal control group; ^B^*p*< 0.05 compared with the BMF group; ^C^*p*< 0.05 compared with the CCCP group; ^D^*p*< 0.05 compared with the flavone group.

Compared with the normal control group, the expressions of PAC-1 and P-selectin of the model control group and CCCP group were increased significantly. Compared with the model control group, the expressions of PAC-1 and P-selectin of flavone group and CSA group were decreased significantly. Compared with the CCCP group, the expressions of PAC-1 and P-selectin of the CCCP + flavone group were decreased significantly. But no significant differences were found between flavone group and flavone + CSA group. It is indicated that the total flavonoids and CSA inhibited the expressions of PAC-1 and P-selectin (Figure 7).

### 3.9. Effect of Total Flavonoids on the Expressions of Bax, Bad, Bid, and Caspase-9 In Vitro

**Figure 8:**
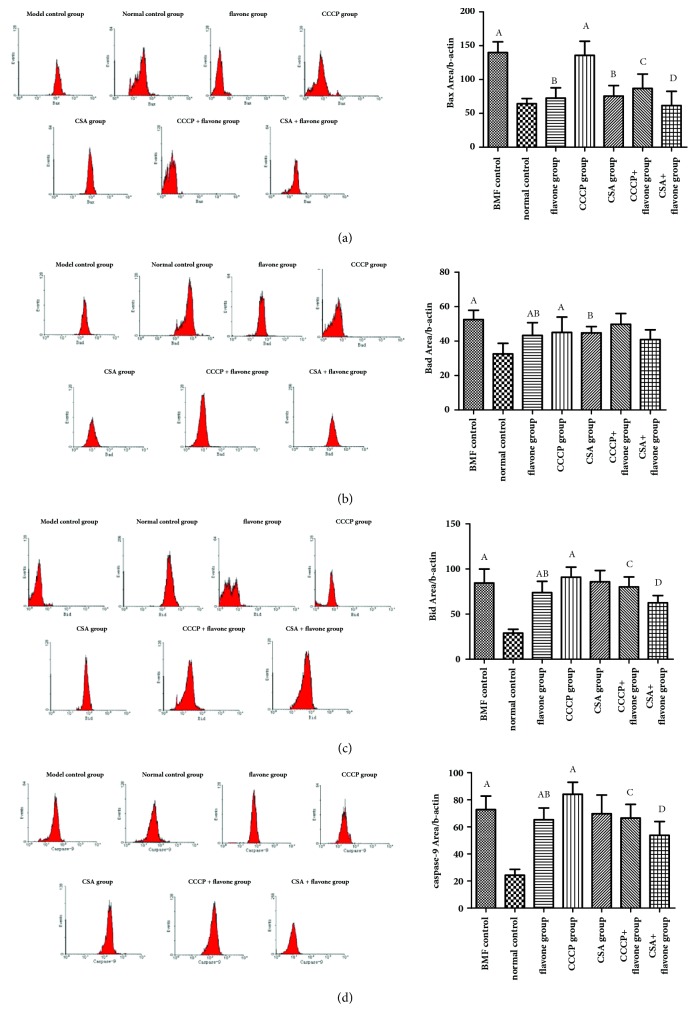
**The expressions of apoptosis markers including Bax, Bad, Bid, and caspase-9. **The washed platelets were prepared and treated with different drug interventions. The expressions of Bax (a), Bad (b), Bid (c), and caspase-9 (d) were detected by flow cytometry. ^A^*p*< 0.05 compared with the normal control group; ^B^*p*< 0.05 compared with the BMF group; ^C^*p*< 0.05 compared with the CCCP group; ^D^*p*< 0.05 compared with the flavone group.

Compared with the normal control group, the expressions of Bax, Bad, Bid, and caspase-9 of model control group and CCCP group were increased significantly; however, total flavonoids inhibited the induction. Besides, CSA inhibited the expressions of Bax and Bad, but did not affect the expressions of Bid and caspase-9. Compared with the CCCP group, the expressions of Bax, Bid, and caspase-9 of CCCP+ flavone group were decreased significantly. Compared with the flavone group, the expressions of Bax, Bid, and caspase-9 of flavone + CSA group were decreased significantly. Therefore, the total flavonoids and CSA could inhibit the expression of the proapoptotic proteins including Bax, Bad, Bid, and caspase-9. The combined application of the total flavonoids and CSA worked even better (Figure 8).

### 3.10. The Morphological Changes of Platelet Apoptosis Recorded by Phase Contrast Microscopy

**Figure 9:**
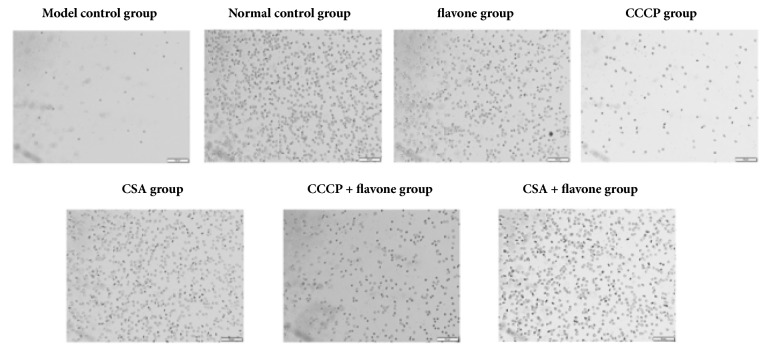
**The morphological changes of platelets (×400). **Changes in the morphology and number of platelet apoptosis from various* in vitro* study groups were recorded by inverted phase contrast microscope.

Platelet microparticles release and platelet retraction are the morphological manifestation of platelet apoptosis and can be observed by electron microscopy (Figure 9). Platelets in normal control group showed scattered distribution, no aggregation or pseudopodia. Most of the platelets were flat disk-like, double-sided slightly convex, and oval-shaped with smooth surface, while a few were small crescent-shaped. Platelets in model control group showed clustered distribution, irregular shapes with droplets, a significant decrease in number, and rough surface. Platelets in flavone group showed scattered distribution, a few aggregation, disk-shape in majority, and stack in layers. Platelets in apoptosis inducer group showed clustered distribution, irregular shapes, a significant decrease in number, and rough surface. Some flat disk-like platelets and a large proportion of irregular crescent-shaped and barrel-like platelets were observed in apoptosis inhibitor group. Platelets in CCCP + flavone group were evenly distributed flat-shaped, disk-shaped, and irregular shaped. Platelets in CSA + flavone group showed scattered distribution, with the majority being flat disk-shaped and the minority being irregular shaped. What is more, compared with the model control group, there were more platelets in the flavone group, the apoptosis inhibitor group, and the CSA + flavone group.

## 4. Discussion

The hemorrhage caused by the thrombocytopenia of bone marrow failure is a very difficult issue in clinic. The bleeding mainly occurs in skins, mucous membranes, and internal organs with repeated attacks. It may even lead to the life-threatening intracranial hemorrhage. Currently drugs for the treatment of bone marrow failure, such as immunosuppressive agents and antithymocyte globulin (ATG), and the treatment of thrombocytopenia, such as glucocorticoid and transfusion of platelets, are difficult in popularization and application worldwide due to the high cost, strong side-effects, or dose-dependent manner. To explore new and effective methods of hemostasis is particularly important. Data statistics showed that traditional Chinese medicine with blood cooling and hemostasis effect works well on the treatment of thrombocytopenia [19]. Our previous studies found that Herba Sarcandrae, one of the traditional Chinese medicines with cooling blood and hemostasis effect, could effectively improve the bleeding of acute bone marrow failure in mice models [20].

In physiological platelets, the PS is distributed on the inside of the lipid bilayer of the cell membrane. The platelets activation by tissue damage or other factors causes externalization of phosphatidylserine PS from the inner membrane layer to the cell membrane surface [21]. PS exposure is a typical apoptotic event in nucleated cells. The amount of PS exposure reflects the proportion of the apoptotic platelets in the total storage platelets. And the proportion of platelet apoptosis directly affects the function of platelets [22, 23]. PS exposure follows the decrease or loss of the mitochondria membrane potential, so it is the sign of the mid stage apoptosis [24]. Because PS exposure is involved in both platelet activation and apoptosis, we further investigated whether the total flavonoids of Herba Sarcandrae also play a role in the platelet activation. Two typical signs of platelet activation, PAC-1 binding and P-selectin, were detected. The small molecule complex of PAC-1 effectively activates procaspase-3 into caspase-3 and induces apoptosis of a variety of tumor cells. PAC-1 activates caspase-3 directly, and caspase-3 activation further activates caspase-8, 9. P-selectin is mainly located in the *α* granules of platelets or in the Weibel-Palade bodies of endothelial cells, with no expression or continuous low expression in the resting time. When platelets are activated, P-selectin is expressed rapidly on the platelet membrane surface through the fusion of *α* granules and platelet membrane. Therefore, PAC-1 and P-selectin are often used as markers of platelet activation [25, 26]. The results showed that the expression of PAC-1 and P-selectin of the apoptosis inhibitor group and the flavonoids group decreased significantly, but still higher than that of the normal control group. Why is the PAC-1 and P-selectin expression not completely inhibited by apoptosis inhibitor and the total flavonoids of Herba Sarcandrae? Are there any other factors that affect their expression? The PAC-1 and P-selectin expression reflects the ability of platelet activation. The effect of the total flavonoids of Herba Sarcandrae on the PAC-1 and P-selectin expression showed that the platelet activation was associated with the platelet apoptosis inhibition of total flavonoids via mitochondrial pathway. It is suggested that other mechanisms may be involved. The specific mechanism needs further study. Since the number of platelets was little, it was only enough and sufficient to run flow cytometry instead of western blotting, but more work, such as western blotting to measure protein expression and Q-RT-PCR to measure mRNA level, is being deployed in the near future.

The Bcl-2 family proteins include antiapoptotic proteins such as Bcl-2 and Bcl-XL and proapoptotic proteins such as Bax, Bad, and Bid. The antiapoptotic proteins and the proapoptotic proteins interact to regulate the stability of mitochondrial structure and function. Both the antiapoptotic and the proapoptotic proteins play important roles in the intrinsic pathway of the platelets' apoptosis. Bak is mainly located in the mitochondria of platelets, while the Bax is located in the cytoplasm of platelets. When the platelets are stimulated by the inducing factors of apoptosis, Bax then translocates from the cytoplasm to the outer membrane of mitochondria under the action of some proteases. The intracellular concentration of Bax also increases. It breaks the balance between the antiapoptotic proteins and the proapoptotic proteins in the Bcl-2 family [27], results in the loss of inhibitory activity of antiapoptotic protein accompanied by platelet mitochondrial dysfunction, and eventually leads to the platelets apoptosis [28]. Caspase plays a vital role in the process of apoptosis. Not only is it the effector of cell disruption, but it also acts like a promoter in activating the apoptosis after receiving the signal. The proapoptotic proteins in the Bcl-2 family act on the mitochondrial membrane of platelets. They cause the decrease of platelet mitochondrial transmembrane potential and the release of cytochrome c. It results in energy metabolic disturbance and the release of apoptosis-inducing protein and further leads to caspases activation. Caspase-9 is an “initiator” in cascade, which plays an important role in the steps of cell apoptosis. The activated caspase-9 works as the initiating factor to activate the downstream caspase, which in turn triggers the caspases cascade, eventually activating the downstream caspase 3 [29]. Therefore, Caspase-9 is activated by mitochondrial apoptotic pathway, and the Caspase-9-mediated mitochondrial cytochrome pathway is called intrinsic apoptotic pathway. The imbalance of Bcl-2 family proteins initiates the proapoptotic or antiapoptotic pathway. We found that the total flavonoids of Herba Sarcandrae reduced the expression of antiapoptotic proteins including Bax, Bad, Bid, and caspase-9 and inhibited the excessive platelet apoptosis via mitochondria pathway. Besides, platelet apoptosis causes the PS exposure, vesicles formation of the cell membrane, and the release of platelet microparticles [30]. In the present study, the morphological and quantitative changes of platelet apoptosis in each group were recorded by contrast microscope. It is indicated that the total flavonoids of Herba Sarcandrae inhibit the excessive apoptosis of platelets through mitochondrial pathway, in which platelet activation is also involved.

In summary, the results of* in vitro* and* in vivo* studies showed that the total flavonoids of Herba Sarcandrae increased the number of platelets, inhibited the expression of apoptosis-accelerating proteins such as Bax, bad, Bid, and caspase-9, inhibited the externalization of PS, decreased the expression of PAC-1 and P-selectin, inhibited the platelet apoptosis, and prolonged the platelet survival time. It is indicated that the total flavonoids improve the number of platelets in bone marrow failure disease by inhibiting the platelet apoptosis via regulating the mitochondrial pathway.

## Data Availability

The data used to support the findings of this study are available from the corresponding author upon request.
